# DNA Input Classification by a Riboregulator-Based Cell-Free Perceptron

**DOI:** 10.1021/acssynbio.1c00596

**Published:** 2022-04-05

**Authors:** Ardjan J. van der Linden, Pascal A. Pieters, Mart W. Bartelds, Bryan L. Nathalia, Peng Yin, Wilhelm T. S. Huck, Jongmin Kim, Tom F. A. de Greef

**Affiliations:** † Laboratory of Chemical Biology and Institute for Complex Molecular Systems, Department of Biomedical Engineering, Eindhoven University of Technology, 5600 MB Eindhoven, The Netherlands; ‡ Computational Biology Group, Department of Biomedical Engineering, Eindhoven University of Technology, 5600 MB Eindhoven, The Netherlands; § Institute for Molecules and Materials, Radboud University, 6525 AJ Nijmegen, The Netherlands; ∥ Wyss Institute for Biologically Inspired Engineering, Harvard University, Boston, Massachusetts 02115, United States; ⊥ Department of Systems Biology, Harvard Medical School, Boston, Massachusetts 02115, United States; # Department of Life Sciences, Pohang University of Science and Technology, Pohang, Gyeongbuk 37673, Republic of Korea; ∇ Center for Living Technologies, Eindhoven-Wageningen-Utrecht Alliance, 3584 CB Utrecht, The Netherlands

**Keywords:** perceptron, genetic classifier, weighted sum operations (WSO), cell-free systems, synthetic genetic networks, synthetic biology

## Abstract

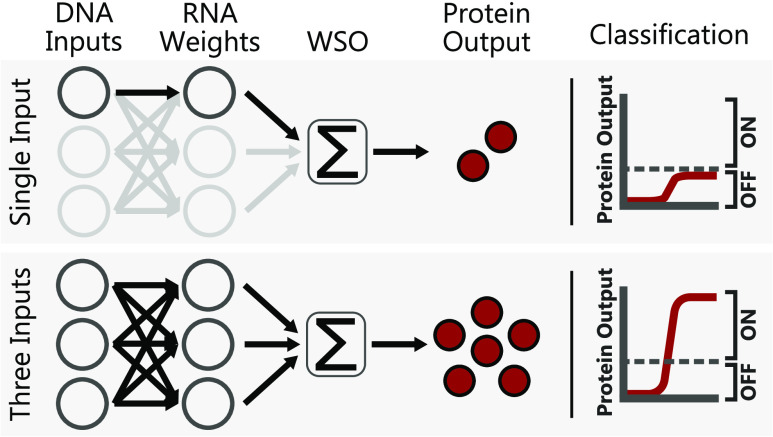

The ability to recognize molecular patterns is essential for the continued survival of biological organisms, allowing them to sense and respond to their immediate environment. The design of synthetic gene-based classifiers has been explored previously; however, prior strategies have focused primarily on DNA strand-displacement reactions. Here, we present a synthetic in vitro transcription and translation (TXTL)-based perceptron consisting of a weighted sum operation (WSO) coupled to a downstream thresholding function. We demonstrate the application of toehold switch riboregulators to construct a TXTL-based WSO circuit that converts DNA inputs into a GFP output, the concentration of which correlates to the input pattern and the corresponding weights. We exploit the modular nature of the WSO circuit by changing the output protein to the *Escherichia coli* σ28-factor, facilitating the coupling of the WSO output to a downstream reporter network. The subsequent introduction of a σ28 inhibitor enabled thresholding of the WSO output such that the expression of the downstream reporter protein occurs only when the produced σ28 exceeds this threshold. In this manner, we demonstrate a genetically implemented perceptron capable of binary classification, i.e., the expression of a single output protein only when the desired minimum number of inputs is exceeded.

## Introduction

Fundamental to the survival of living organisms is their ability to process a wide variety of information, continuously sensing, and, in turn, adapting to their surroundings. Synthetic biology employs the use of naturally occurring biological parts implemented in synthetic networks designed to either mimic existing or introduce novel functionalities to both living cells and artificial platforms.^1−5^ The parallels in information processing between living systems and electronic devices have led to the development of numerous synthetic biological logic circuits inspired by their counterparts in the field of electrical engineering.^6−13^ While the development of such circuits can be seen as a purely scientific exercise,^14−16^ these systems are increasingly being applied toward practical applications such as biomarker recognition for medical diagnostics,^17−21^ the detection of pollutants,^22,23^ and a variety of cell therapies.^24−27^ Herein, the ability to differentiate between various unique biomarkers, as well as combinations thereof, and the subsequent categorization of biomarker patterns into identifiable classes is critical.

**Figure 1:**
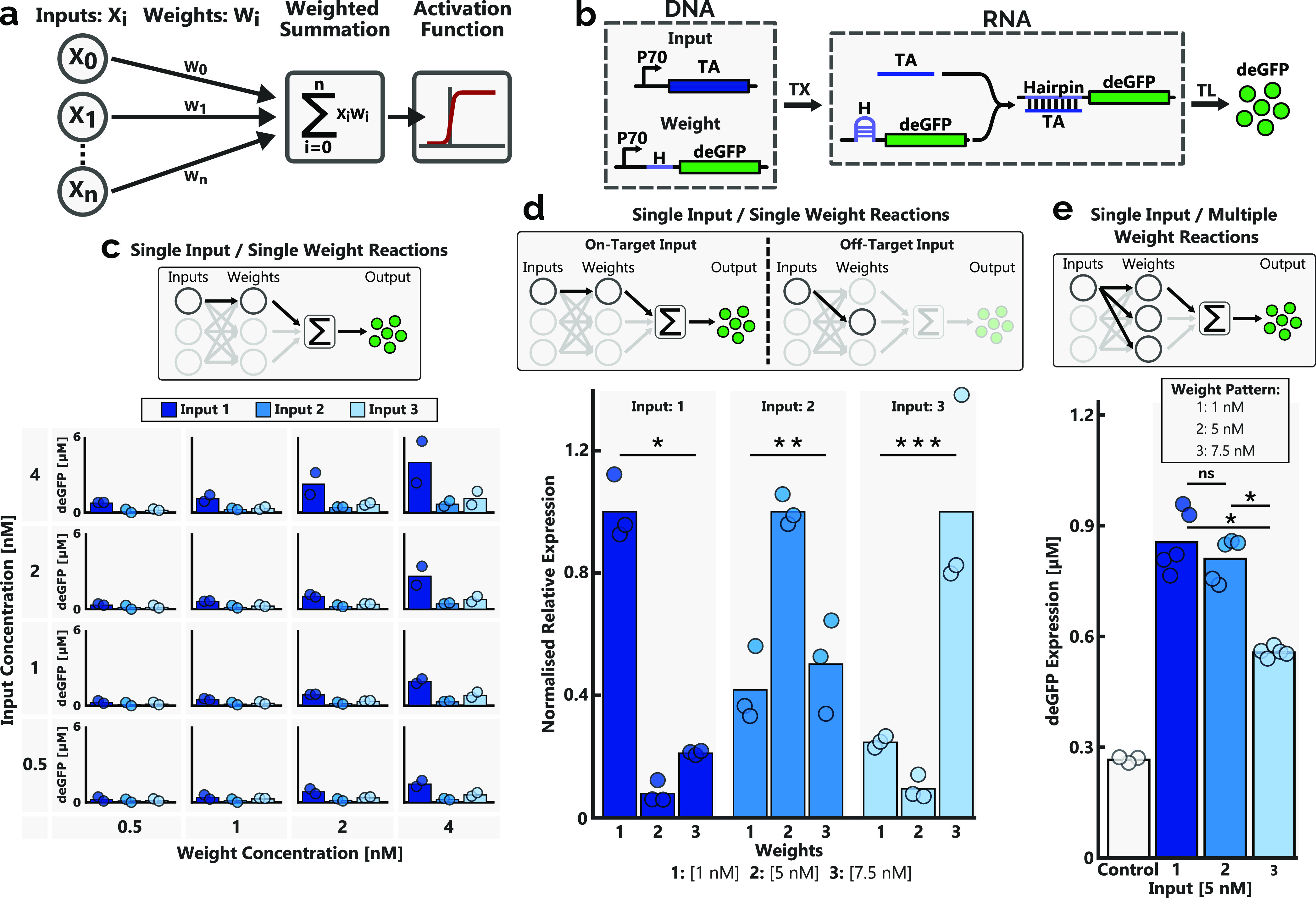
(a) Overview of a perceptron. A range of inputs, each with a unique weight, are summed, with the weighted sum output serving as the input of an activation function, which determines the perceptron output. (b) RNA toehold switch. The transcription of RNA from DNA inputs yields trans-acting RNAs that activate the RNA riboregulator, resulting in the translation of a gene. The DNA templates encoding the trans-acting and transducer RNA strands are designated as the input and weight, respectively. Binding of the trans-acting strand to the toehold of the transducer strand initiates toehold-mediated RNA–RNA strand displacement, whereby the hairpin sequence is unfolded and the ribosome-binding site is exposed, allowing for the expression of the output gene: deGFP. TA and H refer to the trans-acting strand and the hairpin sequence of the transducer strand, respectively. TX: transcription and TL: translation. (c) deGFP expression levels achieved when combining a single input and weight pair, for a range of concentrations. The bar height corresponds to the average of two data points (dots). (d) Normalized relative expression for each input in the presence of on- and off-target toehold switches. Each unique input (5 nM) is exposed to each of the weights (with the concentration corresponding to the weight pattern) in isolation, and the end-point expression level is recorded following a batch expression experiment, as described in the Methods section. The bar height corresponds to the average of three data points (dots). For each input, the data were normalized by dividing the average expression level by the average expression level determined for the on-target (i.e., cognate input and weight pair) reaction. * *p* < 0.001, ** *p* < 0.006, and *** *p* < 0.02. (e) Expression of each unique input (5 nM) in the presence of all three weights (1, 5, and 7.5 nM, respectively, for weights 1, 2, and 3). The control was performed in the presence of the weight pattern, without the addition of any inputs. Input and weight concentrations were optimized to ensure approximately equal expression levels for all inputs. Bars depict the average expression level of at least three experiments (dots). ns = not significant and * *p* < 0.0001. All experiments were conducted using linear DNA constructs in a self-made cell lysate solution under batch conditions, as specified in the Methods section.

Due to their innate ability to function as classifiers, there has been broad interest in the development of synthetic biological neural networks.^28−35^ Perceptrons (Figure 1a), the most basic building block found in neural networks, act as linear classifiers, accepting a range of inputs, each with a corresponding weight, and provide a single binary output.^36,37^ Perceptrons compute a weighted sum of the inputs and their weights, which undergoes thresholding using an activation function to return a single output value corresponding to the classification of the inputs provided. In addition to the simplistic design of a perceptron, it is their ability to accept analog input signals and return a single digital output to perform binary classifications that has led to the prominence of perceptron-based classifiers within the field of synthetic biology.^20,31,33,38^ Furthermore, the possibility to expand the basic perceptron circuit by introducing additional layers and the inclusion of memory devices^39−42^ in the system is pushing the development of biological neural networks that combine molecular pattern classification and molecular data storage.^43^

The versatile and highly programmable nature of DNA and RNA has proven to be exceptionally useful when performing biomolecular computations.^25,43−53^ This widespread usage stems from the ability to rationally design novel DNA and RNA strands, which hybridize predictably with complementary constructs. A majority of the previously published classification networks utilize single-stranded DNA templates and rely upon binding competition and DNA strand displacement reactions.^38,44,54^ However, designs based solely on DNA strand displacement reactions, where DNA strands are prepared synthetically and subsequently combined, lack the information storage capabilities and the broader ability to integrate the circuits into larger, more complex biological systems due to a lack of transcriptional and translational control. Here, we present a genetically implemented perceptron based on *in vitro* transcription and translation (TXTL) reactions.^55−59^

The cell-free genetically implemented perceptron acts as an ON/OFF binary classifier, with the expression of a reporter protein correlating to an ON signal. The final perceptron design consists of two distinct elements: a weighted sum operation (WSO) implemented at the RNA level and a post-translational thresholding reaction. The TXTL-based WSO utilizes toehold switch riboregulators, a class of de novo designed translational riboregulators comprising a cognate pair of RNAs^60−62^ (Figure 1b). A transducer strand is implemented to regulate translation, with a cognate trans-acting RNA serving to modulate its biological activity.^60^ In the context of our implemented WSO, the transducer strands and trans-acting strands serve as the weights and inputs, respectively. Inputs initiate toehold-mediated RNA–RNA strand displacement reactions upon the weights, which have been designed to form a hairpin such that bases in the regions surrounding the ribosome-binding site (RBS) and start codon are sequestered. The expression of genes encoded on the weight construct is thereby inhibited until a complementary input is provided, which first binds to a single-stranded toehold sequence at the 5′ end of the hairpin before completing a branch migration process exposing the RBS and start codon, enabling ribosome binding and subsequent gene expression. The WSO output is determined as the sum of the total concentration of protein expressed by all of the unique input and weight pairs.

The toehold switch technology allows us to couple the output of a WSO at the RNA level to the production of a protein. To realize a TXTL-based perceptron, we implemented an ultrasensitive sink at the protein level based on molecular titration, resulting in a tunable threshold of the WSO output.^63,64^ In this way, the perceptron only presents an output signal (ON state) when the WSO output concentration exceeds that of the threshold set by the concentration of the titrant. By tuning the concentration of the titrant, the classification boundary of the perceptron can be controlled, with higher titrant concentrations requiring greater concentrations of the WSO output to be produced before the ON state of the perceptron is reached. Increasing the WSO output is achieved by increasing the overall concentration of inputs provided to the WSO, either by increasing the concentration of individual inputs or by increasing the number of unique inputs provided. The determination and selection of a specific threshold allow the perceptron to perform a classification of the number of inputs provided to the WSO; only displaying the ON state when a specified minimum number of inputs has been provided.

Here, we present the successful *in vitro* implementation of a novel three-input TXTL-based perceptron, utilizing both RNA- and protein-level regulation technologies to construct a DNA input classifier. Although the addition of transcriptional and translational processes increases the overall complexity of the network design when compared to systems relying solely upon DNA strand displacement reactions,^38,44,54^ it also offers significant advantages. The application of genes encoding protein sequences, as opposed to designing DNA templates solely for strand displacement, enables the up- and downstream usage of these proteins, greatly expanding the functionality of these circuits. Furthermore, the interchangeability of both the DNA inputs and the protein outputs facilitates the implementation of this perceptron in larger complex synthetic genetic networks.

## Results

Construction of the genetic perceptron occurred in several distinct phases, with initial research focused on the development of two WSO networks: the first incorporating the reporter protein directly on the weight templates and the second utilizing the WSO output to regulate the expression of a downstream reporter construct. The latter of these WSO circuits was subsequently used to implement the perceptron via addition of a downstream ultrasensitive sink. The genetic perceptron was designed for *in vitro* implementation, with all experiments occurring under batch conditions using a self-made cell lysate derived from bacteria (*Escherichia coli*, see the Methods section).^55,56^ All of the genetic constructs for the inputs, weights, and the independent reporter were constructed using a Golden Gate assembly-based cloning method.^57^ The DNA constructs used were optimized for RNA stability as well as the efficacy of the input–weight pair, as reported by Pieters et al.^62^ Polymerase chain reactions (PCR) were used to prepare linear DNA templates for use in the experiments (see the Methods section).

### Input–Weight Pair Characterization

Three unique input–weight pairs were constructed to perform WSOs. Initially, to investigate the behavior of input–weight pairs and to investigate their ability to function in a WSO, a fluorescent reporter protein (deGFP) was encoded downstream of the transducer construct hairpin sequence. In this manner, binding of the trans-acting RNA strand (obtained via transcription of the input DNA template) to the toehold of the transducer RNA strand (obtained via the transcription of the weight DNA template) allowed the direct expression of the deGFP reporter (Figure 1b).

The independent expression levels for each of the three inputs, solely in the presence of their cognate weight was determined for a range of both input and weight concentrations (Figure 1c). Despite using identical promoter sites for all inputs and weights, large differences in the reporter end-point concentration of the different input–weight pairs can be seen, revealing disparities in their relative expression strengths. However, the end-point expression levels increased predictably as the concentrations of both the input and weight were increased (Figure S1).

To enable the classification of the number of inputs provided, as opposed to the specific combination of inputs, the end-point expression levels of each input–weight pair should be approximately equal, such that the addition of any input results in an equal and predictable increase in the overall WSO output. Guided by the results provided in Figure 1c, where the output expression for a range of input and weight concentrations is analyzed, a standard input concentration (5 nM) was determined. Subsequently, a weight pattern was determined, wherein the concentration of each of the unique weights was adjusted according to the relative expression strength of each input and weight pair. A weight pattern of weight 1: 1 nM, weight2: 5 nM, and weight 3: 7.5 nM was selected. Upon conducting WSOs, as well as when performing input classification with the perceptron, all weights will be present in concentrations equal to the weight pattern. As such, the orthogonality of the unique input–weight pairs is critical, ensuring that each input is only able to activate the reporter expression of its own complementary weight. The orthogonality of the input–weight pairs was determined by exposing each input to each of the three weights in isolation. For each input, the end-point expression levels of both on- and off-target, where on-target indicates a cognate input–weight pair, were divided by the end-point expression level of the on-target reaction, revealing the relative expression levels of on- and off-target input and weight combinations (Figure 1d). While inputs 1 and 3 show moderate orthogonality, with both off-target weights exhibiting at least a fivefold reduction in their relative expression, input 2 reveals high background expression levels in the presence of off-target weights, albeit approximately halved with regard to the on-target expression level.

The prior determination of the orthogonality of the input–weight pairs was conducted in isolation, as opposed to the combination of weights provided by the weight pattern applied during WSO experiments. Therefore, each of the inputs was individually exposed to the weight pattern, demonstrating the ability of each input to activate reporter expression while simultaneously being exposed to off-target weights (Figure 1e). Furthermore, the end-point expression levels of the reporter highlighted the efficacy of the chosen input concentration and weight pattern, with each input eliciting satisfactory levels of reporter protein expression, albeit with input 3-induced expression presenting reduced end-point expression levels.

### Direct-Expression WSO

**Figure 2:**
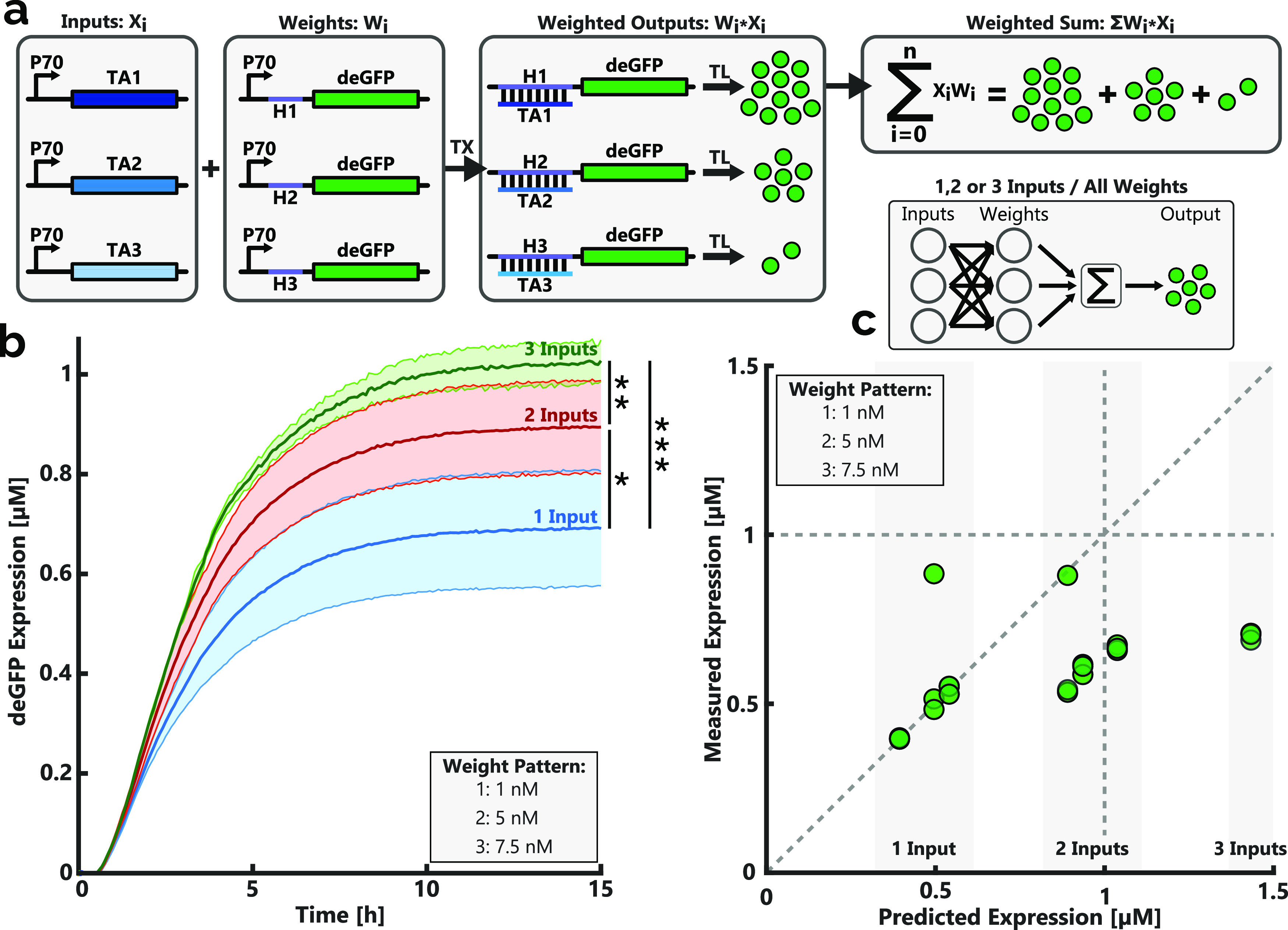
(a) Schematic overview of a weighted sum operation (WSO) utilizing three unique inputs and their corresponding weights. Inputs take the form of DNA constructs encoding trans-acting RNAs and can either be added or omitted from experiments. Transducer strand encoding DNA constructs form the weights, with all three switches being present during all experiments. Addition of an input to the system will result in the expression of deGFP contributing to the total sum of deGFP produced. The increase in expression because of input addition is dependent on the weight concentration. TA and H refer to the trans-acting strand and the hairpin sequence of the transducer strand, respectively. TX: transcription and TL: translation. (b) Average expression curves of all single-, double-, and triple-input reactions. Bold lines indicate the average of these experiments, with the shaded regions indicating the standard deviation. For each unique combination of inputs, (3× single input, 3× double input, and 1× triple input) a minimum of two batch experiments were conducted, as described in the Methods section. All inputs provided were 5 nM in concentration, and all three weights were present during all reactions with the weight pattern as follows: weight 1: 1 nM, weight 2: 5 nM, and weight 3: 7.5 nM. The expression levels were recorded every 5 min for a duration of 15 h. The average expression level per input class was determined as the average of all reactions conducted with the specified number of inputs for each class. * *p* = 0.02, ** *p* < 0.02, and *** *p* = 0.001. Figure S2 specifies the expression curve for each of the unique input combinations. (c) Model-predicted expression levels plotted against experimentally determined values. Model parameters α, β, and γ were fit to single-input end-point expression data and subsequently used to predict the system behavior for additional inputs (see Supporting Methods 1). Expression data were obtained from batch experiments conducted as per the Methods section. All inputs were provided at a concentration of 5 nM, with all weights present according to the specified weight pattern. At least three batch experiments were conducted for each unique combination of inputs.

Completing the development of the WSO, combinations of inputs were provided to reactions comprising all three weights in their respective concentrations. Each additional input provided is expected to supplement the overall pool of deGFP expressed, with the total expressed concentration serving as a readout of the WSO (Figure 2a). With the weight concentrations scaled relative to the expression strength, it is expected that each additional input will increase the total expressed concentration by an identical amount, with the number of inputs provided determining the expression level. Expression data revealed that the average expression levels for single-, double-, and triple-input reactions could be differentiated; however, the increases between these averages were not uniform (Figure 2b). The additional resource burden resulting from the addition of each additional input could explain the nonlinear increase in expression levels.^65−67^ Based on the reporter expression levels for single-input reactions, a rudimentary model was developed (Supporting Methods 1). Each input added to a WSO is analogous to adding an additional term to the summation, with a value equal to the input concentration (*x*) multiplied by the weight concentration (*w*). By further multiplying each term with a scaling factor, representing biological processes such as transcription, translation, and RNA binding, it is possible to predict expression levels. To determine the scaling factors α, β, and γ, the rudimentary model function (eq 1) was fit to the single-input expression data of the WSO experiments (Figure 2c, Supporting Methods 1).
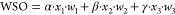
1

Due to the simplicity of the model, with a single variable for each of the input–weight pairs, the model fit aligned with the average expression determined from a series of triplicate experiments. Implementing the model optimized scaling factors, predictions of the expression levels for multi-input experiments were made. In each case, the model prediction exceeds the experimentally determined expression levels, reiterating the nonlinear behavior of the WSO upon increasing the number of inputs. The model assumes expression levels identical to those achieved with single-input reactions, regardless of the burden placed on the system via the introduction of additional inputs.

### Coupling WSO to a Downstream Network

**Figure 3:**
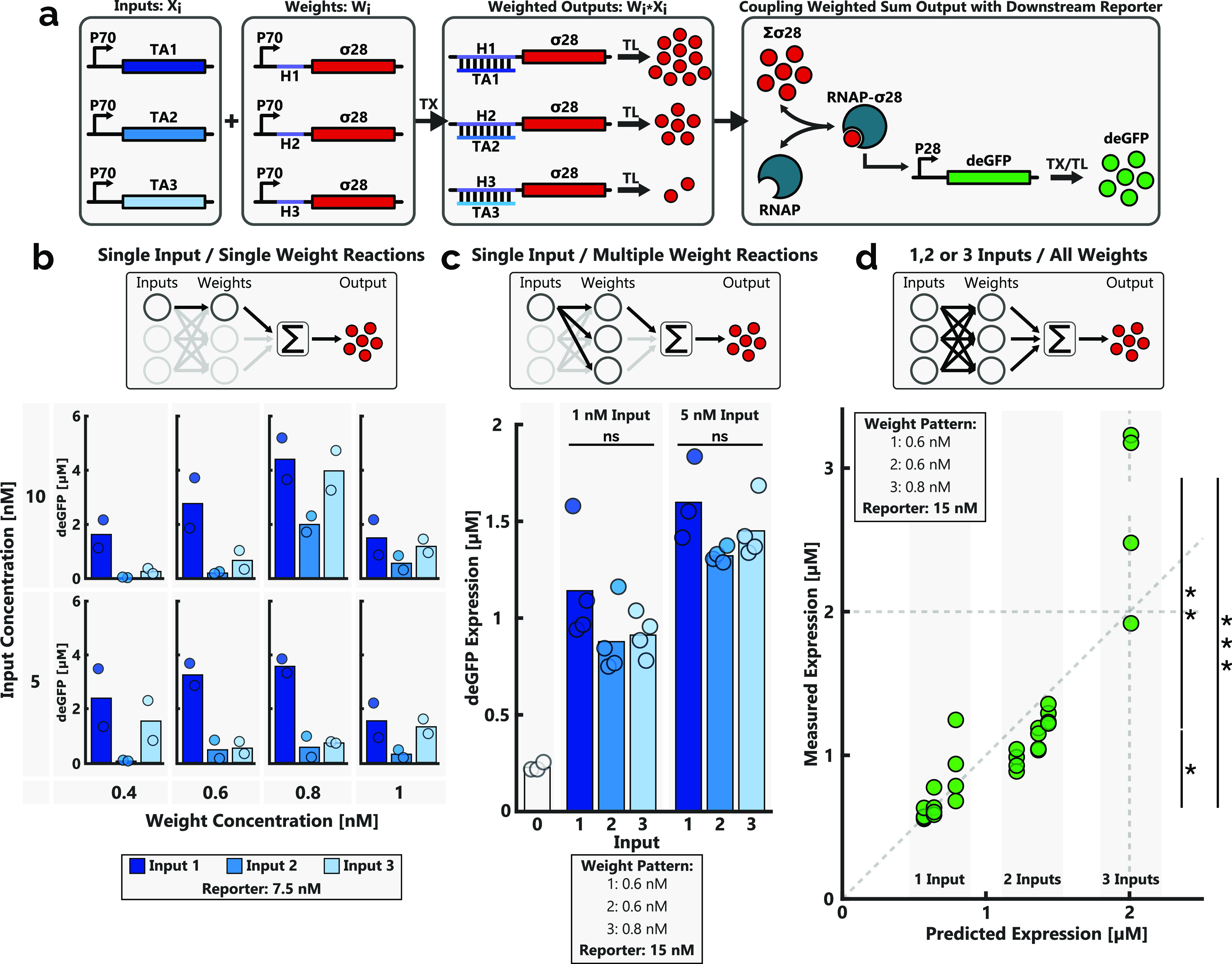
(a) Coupling of the WSO is achieved by replacing the WSO output with the σ^28^ sigma factor. Here, the WSO output is the total amount of produced σ^28^, which serves as an activator for the downstream expression of the deGFP reporter protein. TA and H refer to the trans-acting strand and the hairpin sequence of the transducer strand, respectively. TX: transcription and TL: translation. (b) End-point deGFP expression levels of the reporter protein for each of the unique input and weight pairs, across a range of concentrations. The reporter DNA template concentration was 7.5 nM. Bars indicate an average measurement of two reactions (dots). (c) End-point expression levels of each unique input in the presence of all three weights (0.6, 0.6, and 0.8 nM for weights 1, 2, and 3, respectively). Two input concentrations (1 and 5 nM) were tested, and a reporter template concentration of 15 nM was used. In each case, the bar represents the average expression of two individual experiments (dots). A Grubbs outlier test was used to eliminate a statistical outlier present in the 5 nM input 1 data set. ns = not significant. (d) Experimentally determined deGFP expression levels plotted against the model-predicted values (see Supporting Methods 2). The rudimentary model parameters were fit to single-input end-point expression data and subsequently applied to predict expression levels for multiple-input reactions. Experimental expression data were acquired via batch reactions where the end-point expression levels of all unique combinations of inputs (5 nM) were determined in the presence of all weights (corresponding to the specified weight pattern) and the reporter construct (15 nM). At least three batch experiments were conducted for each unique combination of inputs. * *p* < 0.0001, ** *p* < 0.007, and *** *p* < 0.02. All experiments were conducted using linear DNA templates in a self-made cell lysate solution under batch conditions, as specified in the Methods section.

Computationally, it is possible to classify single-, double-, and triple-inputs from the results of the described WSO; however, a biological implementation of a perceptron or a classifier using this network is nontrivial. Instead, to facilitate a gene-based classifier network, the deGFP reporter protein serving as the WSO output was exchanged for the *E. coli* sigma factor σ^28^.^68^ In doing so, the WSO output could be coupled to the expression of downstream genes while also allowing thresholding of the WSO output, providing perceptron-like behavior. To construct the σ^28^-coupled WSO network, each of the weight constructs was altered to encode for the σ^28^ gene. Additionally, a reporter construct was designed, coding for the deGFP reporter protein, which was placed under transcription control of the P28a promoter. In the resulting system, σ^28^ expressed as the WSO output competes with the σ^70^ present in the cell lysate for binding with core RNA polymerase (RNAP), each forming their respective holoenzymes necessary for the transcription of genes.^69^ Subsequently, the σ^28^ holoenzyme can initiate transcription of the reporter construct, providing a fluorescent readout, which is both dependent on and correlated to the outcome of the WSO (Figure 3a).

Each of the inputs was exposed to its complementary weight in isolation across a range of input and weight concentrations to confirm the downstream coupling pathway while also indicating the relative expression strengths of each of the redesigned input–weight pairs (Figure 3b). Again, each input–weight pair possessed a unique expression strength while displaying predictable responses to increases in the weight concentration. The weight concentrations applied here were significantly reduced compared to the previous system, with end-point expression levels decreasing at weight concentrations above 0.8 nM in almost all cases. Similarly, the expression levels achieved using an input concentration of 5 nM were often comparable to those achieved using a higher 10 nM input concentration, indicating diminishing returns when using elevated DNA input concentrations (Figure S3). Guided by data from Figure 3b, an updated weight pattern was determined so as to ensure similar expression levels for each input: weights 1 and 2: 0.6 nM and weight 3: 0.8 nM. Exposing each input individually to the aforementioned weight pattern resulted in statistically equal levels of reporter expression (Figure 3c). Here, an input concentration of 5 nM resulted in superior expression levels when compared to 1 nM input concentrations, further indicating optimal expression conditions when using 5 nM inputs.

Conducting WSOs with the coupled network showed predictable increases in deGFP expression levels when increasing the number of inputs provided (Figure 3d). Appending the rudimentary model, such that the WSO output is used as an activator for the expression of the reporter, once more enables the computational prediction of expression levels for multiple inputs. As with the direct-expression WSO, the model was first fit to expression data from single-input experiments to determine the system-specific scaling factors (see Supporting Methods 2). Hereafter, predictions of the expression levels were compared to the experimentally acquired results of multi-input reactions. Despite predicting higher expression levels, the model predictions closely replicate the experimentally determined expression levels, which were marginally lower than predictions in all cases. The clear grouping of single-, double-, and triple-input expression levels allows the classification of the system into single-, double-, or triple-input classes by analyzing only the reporter expression levels.

### Genetically Implemented Perceptron

**Figure 4:**
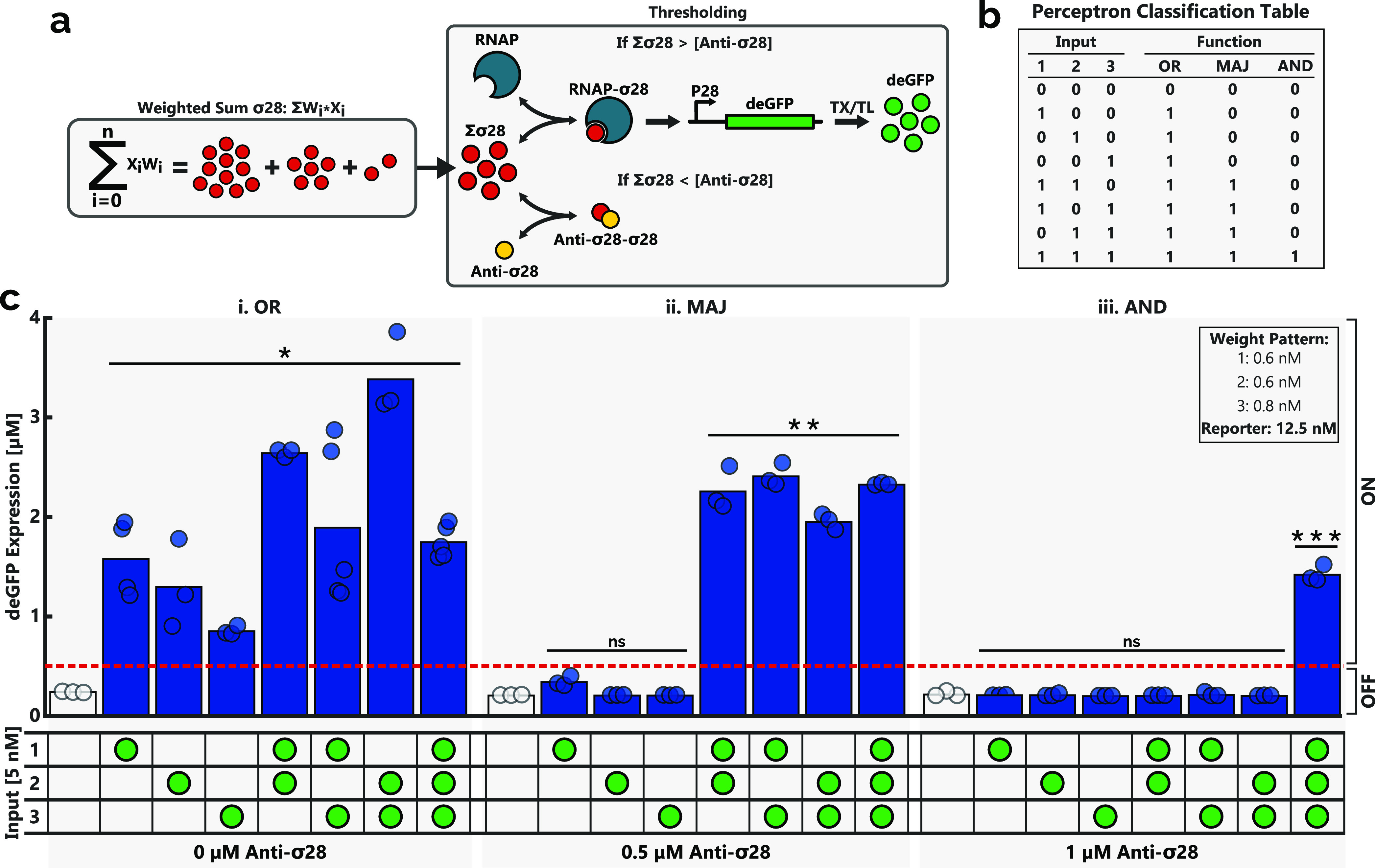
(a) Application of a thresholding mechanism, in the form of anti-σ^28^ allows for the creation of a TXTL-based perceptron. The anti-σ^28^ competitively binds to the σ^28^ produced during the WSO, inhibiting it from activating the expression of the reporter protein deGFP. Altering the concentration of the anti-σ^28^ changes the WSO output concentration required for reporter expression to occur. (b) For each unique input combination, the desired perceptron output or target can be determined for a range of logic functions. A ‘0’ target indicates no expression of the reporter and a ‘1’ target indicates reporter expression. (c) Variations in the anti-σ^28^ concentration allowed for the experimental realization of each of the classification functions. Expression is said to be ‘OFF’ when the expressed deGFP concentration is below 0.5 μM. The inputs (5 nM) added to each of the batch reactions are indicated by the green circles. All three weights were present in each reaction with the following weight pattern: weights 1 and 2: 0.6 nM and weight 3: 0.8 nM. Additionally, a 12.5 nM deGFP reporter construct was added to each reaction. The end-point expression was determined following a batch reaction performed as described in the Methods section. The negative control (white bar) was obtained in the presence of all weights and the reporter but lacked any of the inputs. The height of the bars corresponds to the average of at least three experiments (dots). Significance was determined based on a one-sided t-test to determine if the mean of each unique reaction was significantly greater than the threshold value of 0.5 μM. * *p* < 0.05, ** *p* < 0.003, *** *p* < 0.002, and ns = not significant.

To demonstrate a TXTL-based perceptron, thresholding of the WSO output was implemented. The binding of σ^28^ with the core RNAP was inhibited via the addition of the anti-σ^28^ protein to the system, which competitively binds to free σ^28^. Furthermore, the addition of anti-σ^28^ can also promote the dissociation of σ^28^ from the core RNAP.^70^ The addition of sufficient anti-σ^28^ is therefore able to inhibit the expression of the deGFP reporter (Figure 4a). By tuning the concentration of anti-σ^28^ supplied to each reaction, it is possible to tune the perceptron threshold such that the expression of the reporter requires at least one, two, or three inputs. As shown in Figure 4b, this is analogous to implementing “*OR*”, “*MAJORITY*”, and “*AND”* functions with the perceptron. Furthermore, it highlights the ability of the perceptron to act as a molecular classifier, which can distinguish the number of inputs, only returning an output when the number of provided inputs matches the desired logic function. Experimentally, each of these classification functions was realized by varying the concentration of anti-σ^28^ added to each of the reactions (Figure 4**c**). As expected, by omitting the anti-σ^28^, deGFP reporter expression occurred in all cases where at least a single input was provided, correlating with the desired *OR* function. Increasing the anti-σ^28^ concentration to 0.5 μM resulted in reactions with fewer than two unique inputs being unable to exceed 0.5 μM deGFP expression, the minimum expression level required for the network output to qualify as ON. At the same anti-σ^28^ concentration, reactions with at least two inputs (i.e., the majority of inputs being present) were able to express sufficient deGFP to classify the output signal as ON and thereby realize the *MAJORITY* function. Upon increasing the anti-σ^28^ concentration to 1 μM, only the three-input reaction was able to express over 0.5 μM deGFP to provide the ON signal, in accordance with the *AND* function. The concentration of anti-σ^28^ required to demonstrate each of these classification functions was found experimentally, initially by increasing the concentration of anti-σ^28^ added to three-input reactions (Figure S4), providing an upper limit for the inhibitor concentration beyond which no deGFP expression would occur regardless of the number of inputs. Hereafter, the concentration was lowered incrementally until the one- and two-input reactions were successfully inhibited. Due to the variations in expression strength for each of the unique input and weight pairs, it was possible to achieve scenarios wherein only one or two of the single-input reactions was successfully repressed (Figure S5), highlighting the importance of adjusting the weight pattern such that each of the input and weight pairs expressed similar levels of the reporter, thereby minimizing the effort required to find a suitable anti-σ^28^ concentration.

## Discussion and Conclusions

With this work, we demonstrate a TXTL-based perceptron, utilizing toehold switch regulators to compute the necessary weighted sum operations. Central to the behavior of a perceptron is the ability to perform a binary classification of the given inputs by applying an activation function to the output of the WSO; in our case, a thresholding function. This was achieved experimentally via the introduction of a σ^28^ inhibitor, the concentration of which determined the classification boundary of the perceptron. The mathematical equivalent hereof is the adaptation of the bias term within the perceptron threshold function (eq 2).
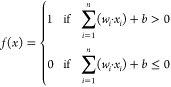
2

From eq 2, it follows that the addition of anti-σ^28^ to the reactions functions as the implementation of the bias term ***b***. Without the inclusion of this term, the weighted sum output of any input ***x*** with its corresponding weight ***w*** results in a greater than zero outcome, and thus a “***1***” or ON output of the thresholding function ***f***. To obtain three unique classifiers, the bias term of each perceptron was altered by varying the anti-σ^28^ concentration, whereby the overall output of the WSO required to induce an ON state output was also altered. As such, we have demonstrated the ability to engineer a TXTL-based perceptron, capable of conducting three unique classifications via changes in the inhibitor concentration.

Unique to our genetic perceptron design is the incorporation of both transcription and translation reactions. Alternative designs have shown the effectiveness of toehold strand displacement reactions for the conducting of WSOs; however, such approaches are limited with regard to the outputs they can offer. The addition of translation enables the production of fluorescent proteins for the straightforward readout of the classification. Alternatively, when applied to theragnostics, the system can be utilized to directly couple the classification of biomarkers with the production of therapeutics specific to the detected input pattern, providing immediate personalized treatment for each unique patient.^71^ Furthermore, the TXTL-based approach presented here offers the ability to incorporate the perceptron design into larger genetic networks, in particular, due to the versatility of the input and output constructs. By substituting the current promoter site, any upstream networks expressing transcription factors or transcription inhibitors could be used to (in-)activate the transcription of the DNA inputs. Similarly, when incorporating the perceptron into larger complex networks, the fluorescent reporter protein used here to quantify the perceptron output can be replaced with an alternative, functional protein. Similarly, both the WSO output and the reporter output protein can be substituted for a functional RNA sequence.^72^ The broad range of functionalities available to RNA, including the activation and repression of transcription, the control of riboswitches, and fluorescence labeling via aptamers such as spinach,^73^ allow for the design of transcription-only networks. In doing so, the metabolic burden of the system can be reduced while retaining the ability to couple multiple perceptrons, either using RNAs to serve as the input of a downstream perceptron, binding directly to the weights, or via the use of RNA transcriptional activators.^74−76^

In addition to the versatility of our gene-based approach, the application of RNA toehold reactions allows for the introduction of additional input–weight pairs, with the highly programmable nature of DNA ensuring that orthogonality between the unique pairs is maintained. However, the inherent variability in the transcriptional and translational efficiency between unique input and weight pairs makes the correct identification of distinct input classes a nontrivial exercise. In an attempt to minimize the variations in transcriptional efficiency, the DNA constructs used within this study were kept identical outside of the specific input and weight sequences; however, due to the placement of the RBS and start codon within the hairpin of the weight construct, this was difficult to achieve on a translational level, an issue further compounded by the inherent differences in the kinetics of each of the unique toehold–switch pairs. Moreover, with each additional input–weight pair, the resource depletion increases, reducing the overall output of the system,^67^ in turn, minimizing the differences in the overall output concentration between classes. In both cases, careful tuning of the weight pattern to ensure near-identical expression levels for each pair can minimize this issue, while reducing the individual expression levels of all input–weight pairs can further reduce the resource burden. Furthermore, continuous flow reactions can be implemented to provide sufficient transcription and translation resources over prolonged durations.^62,77^

Ultimately, the perceptron is regarded as a basic building block of larger neural networks that are capable of learning. The cell-free, genetically implemented perceptron presented in this work can implement a linear classification boundary between two groups of input sets, distinguishing between input sets with the desired minimum number of inputs and those without. Manually, we were able to alter the position of this classification boundary by varying the anti-σ^28^ concentration. However, for our perceptron to be capable of learning, a means of autonomously altering the weight pattern based on the perceptron output and the given inputs is required. A combination of experimental and computational methods has previously been used to train synthetic networks.^33,35^ Here, the experimental output was compared to a desired target output, with the difference being used to determine the updated weight pattern for each iteration, until the difference between the desired and measured outcomes is minimized. In a similar manner, continuous flow reactions could be implemented to update the weight pattern at set intervals following the real-time monitoring of the output concentration, providing semi-autonomous perceptron behavior. Alternatively, to achieve a fully biological implementation of the perceptron, an enzymatic approach can be applied, where specific RNA sequences are amplified or cleaved based on the current perceptron output.^78^ By engineering such sequences to competitively bind to the transducer strands, and in doing so, blocking the toehold site, the effective weight pattern can be adapted without altering the concentration of weights added to the system. The current implementation of the perceptron presented here lacks this learning functionality, instead relying on the manual tuning of the classification threshold. As such, our perceptron functions as a tunable classifier, targeted toward implementation within larger complex genetic networks. However, the versatile design of our system should allow for the introduction of learning, thus enabling the development of multilayer neural networks capable of responding to perturbations of the environment while retaining functionality. Furthermore, autonomous learning alleviates the efforts required to optimize the perceptron weight patterns, facilitating the usage of a single system toward multiple applications, wherein the system trains itself to function on a per-application basis.

## Methods

### DNA Template Preparation

DNA constructs were assembled via the Golden Gate Assembly (GGA) methods, using overlapping sequences previously described by Sun et al.^57^ The assembly vector used (pBEST vector) was gifted by Richard Murray and Vincent Noireaux (Addgene plasmid #45779). The vector was adapted for GGA using Gibson assembly (NEB Gibson Assembly Master Mix) with PCR products of the vector (NEB Phusion High-Fidelity DNA Polymerase) using primers pBEST_GA_1_F, pBEST_GA_1_R, pBEST_GA_2_F, and pBEST_GA_2_R (Table S1). The transducer (switch) and trans-acting (trigger) sequences used were obtained from previous studies by the group of Dr. P. Yin (switches 1 and 2 are unpublished, and switch 3 is identical to switch 1 of the second generation of switches published by Green et al.^60^). All switch sequences are around 67 nucleotides in length; however, triggers 1 and 2 are significantly shorter (55 nucleotides) than trigger 3 (105 nucleotides), which was designed to include a hairpin architecture to enable NOT-gate computations. This additional functionality was not investigated within the scope of this study, with trigger 3 being applied in an identical manner to triggers 1 and 2. Transcription of all inputs and weights was regulated by a σ^70^sigma factor specific P70a promoter derived from the lambda phage,^79^ which was edited to remove the OR3 binding site as per the sequence provided by Richard Murray and Vincent Noireaux (Addgene plasmid #45779). σ^28^ transcription was regulated using an *E. coli* ptar promoter sequence,^80^ where the noncritical −44 to −37 region was adapted from the wild type to match the sequence provided by Richard Murray and Vincent Noireaux (Addgene plasmid #45780). All other additions to the vector construct, such as promoters, coding sequences, and terminators, were ordered as gBlocks from IDT or amplified from the pBEST vector using PCR. Gene sequences can be found in Table S1, alongside the complete vector sequence of the σ^28^-producing weight 1 construct. PCR products of all of the required components were purified using the QIAquick Gel Extraction Kit (Qiagen) and equimolar amounts of each were added to GGA reactions together with BsaI-HF (NEB), T4 ligase (Promega), and T4 ligase buffer (Promega). The GGA reactions were conducted in a thermocycler following a standard GGA protocol.^81^ Completed vectors were transformed into NovaBlue cells (Merck). Plasmid purification was performed using the QIAprep Spin Miniprep Kit (Qiagen), and DNA sequences were confirmed using Sanger sequencing. For TXTL reactions, linear DNA templates were used throughout. The prepared vectors were linearized and amplified by PCR (Phusion High-Fidelity DNA Polymerase, NEB) using the pBEST_LinL2_F and pBEST_LinL2_R primers (Table S1). Final purification of the DNA templates was conducted using the QIAquick PCR Purification Kit (Qiagen).

### Preparation of TXTL Reactions

The cell lysate, energy mixture, and amino acid solutions used to prepare the TXTL reaction solution were prepared identically to the protocol described by Pieters et al.^62^ To prepare a master mix of all reaction components, excluding the DNA templates, the following were combined (with the final concentration given in brackets): cell lysate (33% of the final reaction volume), energy mixture^55^ (7.1% of the final reaction volume), a constant distribution amino acid solution^82^ (37.5 mM), magnesium l-glutamate (10 mM), potassium l-glutamate (40 mM), PEG-8000 (2%), and GamS protein (3 μM). When combined, this solution comprised 69% of the total reaction volume, with the linear DNA templates supplemented with Milli-Q water accounting for the remaining 31%. For experiments requiring the use of anti-σ^28^, the required concentration thereof was incorporated into 31% of the remaining volume alongside the DNA templates. The anti-σ^28^ was purchased (Gentaur) in purified form following expression by an *E. coli* host, and was provided with an N-terminal 10× His-tag and a C-terminal Myc-tag.

### Batch Reactions

All experiments presented here were performed as batch TXTL reactions, with a total volume of 9.5 μL. A total of 10 μL of each TXTL reaction was prepared (6.9 μL of the master mix solution and 3.1 μL of the DNA template solutions), of which 9.5 μL was transferred to a 384-well, round-bottom, NBS-treated microplate (VWR). A Synergy H1M (Biotek) plate reader was used to incubate the microplate at 29 °C, while the deGFP expression was measured (excitation: 470 nm, emission: 510 nm) every 5 min, for a duration of 16 h. The plate reader was calibrated using a titration range of purified deGFP protein. The concentration of the expressed reporter was calculated using the calibration data.

### Statistical Analysis

A two-tailed Welch’s *t*-test was used to compare data sets for which the hypothesis was that the data sets were significantly different (Figures 1d, 2b, and 3d). A one-way ANOVA test was used in cases where the hypothesis was that the data sets would be equal (Figures 1e and 3c). A one-sided *t*-test was used to determine if the mean of a data set was significantly greater than a given threshold value (Figure 4c).

## Supporting Information Available

Figures S1–S5, overview of DNA sequences used (Table S1), models used in the work (Supporting Methods 1 and 2), and complete plasmid sequence used for DNA construct preparation (PDF)
