# Supplementary material for: DNA Input Classification by a Riboregulator-Based Cell-Free Perceptron
Source: ACS Synth Biol. 2022 Apr 5;11(4):1510–20. doi: 10.1021/acssynbio.1c00596 (PMC9016768; doi:10.1021/acssynbio.1c00596)
Supplement: Supplementary file 1 — sb1c00596_si_001.pdf [file sb1c00596_si_001.pdf]

Supplementary Information

DNA Input Classification by a Riboregulator-Based Cell-Free Perceptron

Ardjan J. van der Linden[†,‡], Pascal A. Pieters[†,‡], Mart W. Bartelds[¶], Bryan L. Nathalia[‡], Peng Yin[§], Wilhelm T. S. Huck[¶,*], Jongmin Kim[ǁ,*] and Tom F. A. de Greef[†,‡,¶,#,*]

[†]Laboratory of Chemical Biology and Institute for Complex Molecular Systems, Department of Biomedical Engineering, Eindhoven University of Technology, P.O. Box 513, 5600 MB Eindhoven, The Netherlands

[‡]Computational Biology Group, Department of Biomedical Engineering, Eindhoven University of Technology, P.O. Box 513, 5600 MB Eindhoven, The Netherlands

[§]Wyss Institute for Biologically Inspired Engineering, Harvard University, Boston, MA 02115, USA; Department of Systems Biology, Harvard Medical School, Boston, MA 02115, USA

[ǁ]Department of Life Sciences, Pohang University of Science and Technology, 77 Cheongam-ro, Pohang, Gyeongbuk 37673, Republic of Korea

[¶]Institute for Molecules and Materials, Radboud University, Heyendaalseweg 135, 6525 AJ, Nijmegen, The Netherlands

#Center for Living Technologies, Eindhoven-Wageningen-Utrecht Alliance, Eindhoven, the Netherlands

Corresponding Authors:

*E-mail: jongmin.kim@postech.ac.kr, w.huck@science.ru.nl, and t.f.a.d.greef@tue.nl

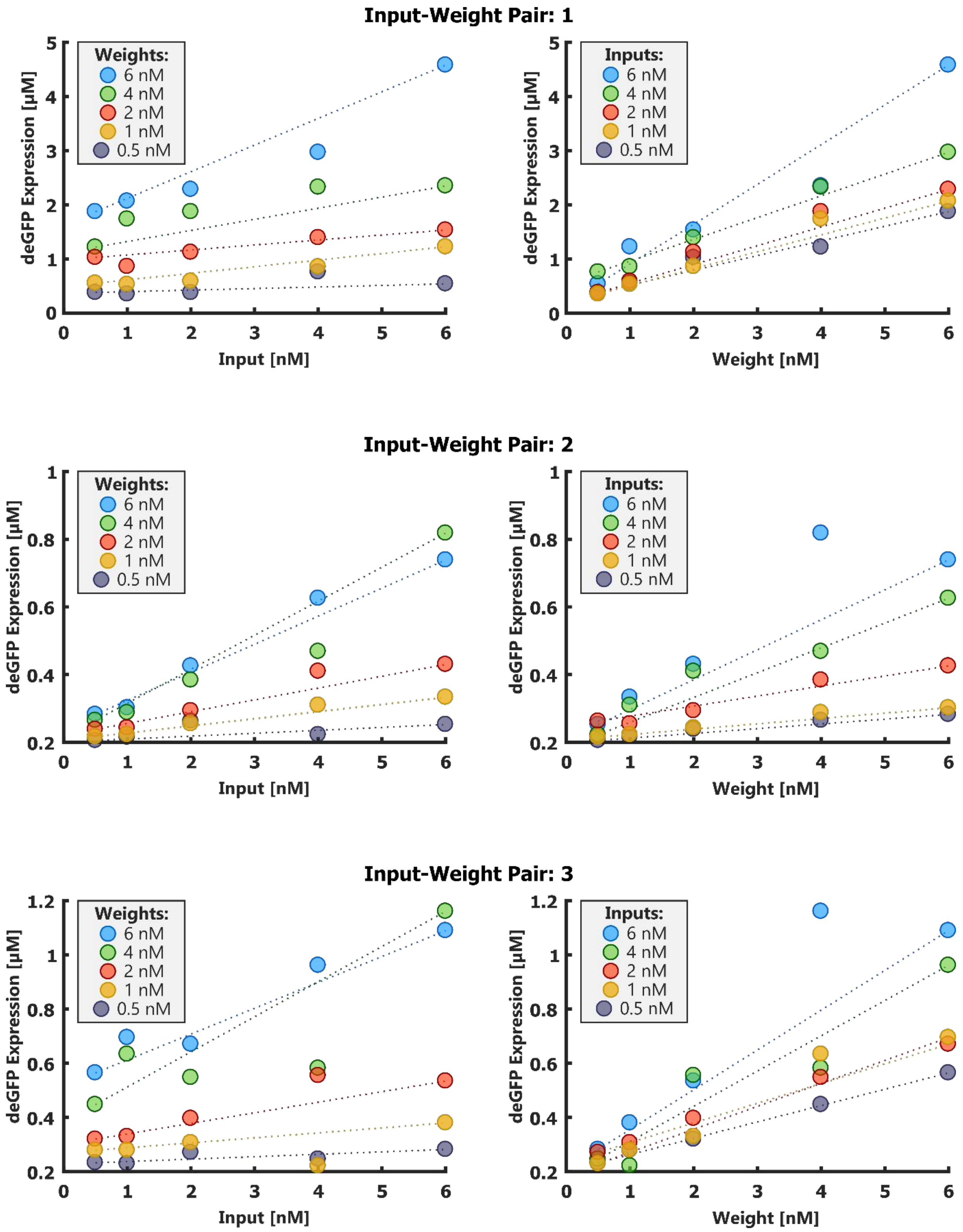

Figure S1: Linearity of the endpoint expression levels when varying the concentrations of either the input or weight for each of the unique input-weight pairs. Dashed lines are the linear guides connecting first and last measurement points of each input/weight range.

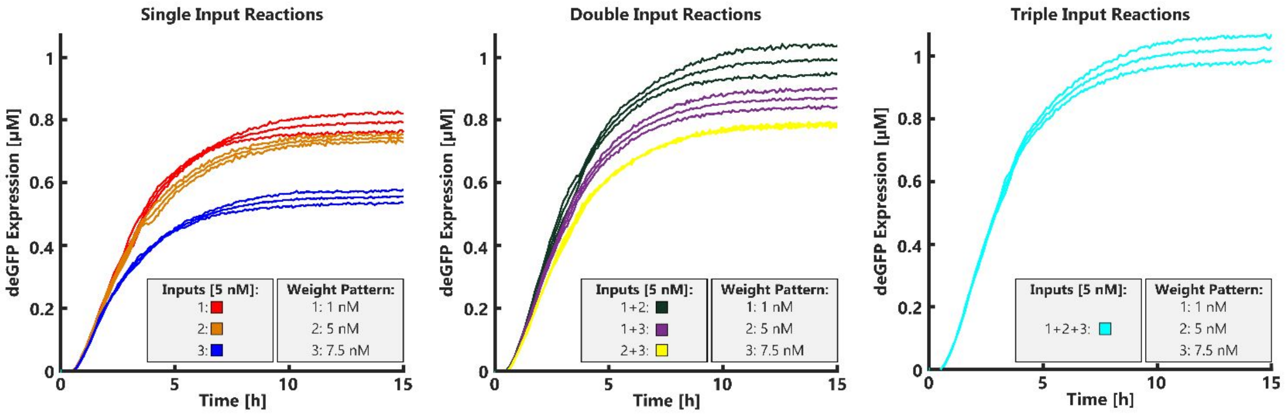

Figure S2: deGFP expression curves for each of the unique reaction used within figure 2b. Here each of the unique input combinations has been individually resolved. The batch experiments were conducted as described in the Methods. All inputs provided were 5 nM in concentration, and all three weights were present during all reactions with the weight pattern: weight 1: 1 nM, weight 2: 5 nM, and weight 3: 7.5 nM. The expression levels were recorded every 5 minutes for a duration of 15 hours.

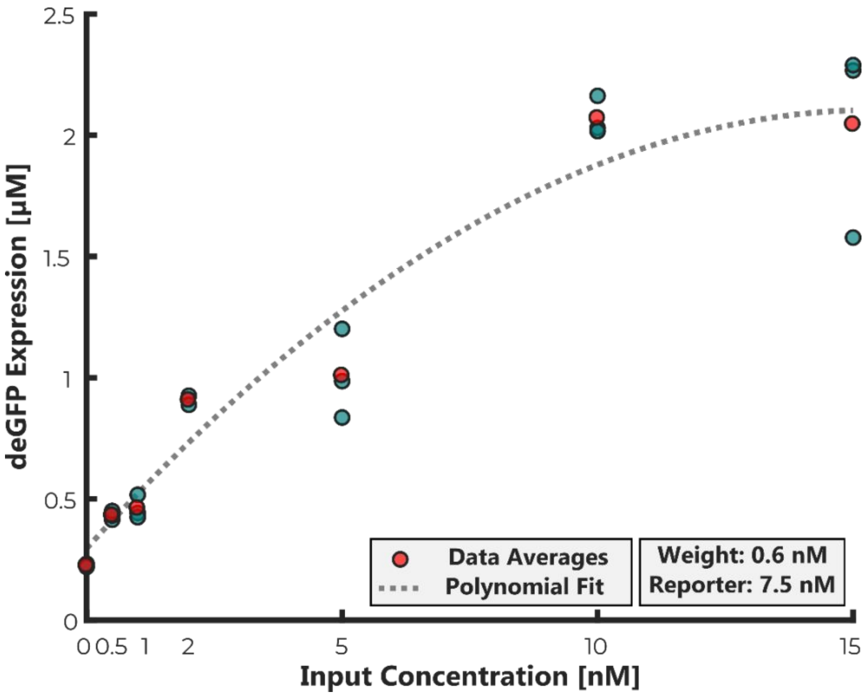

Figure S3: Transfer function of input/weight pair 1. All experiments were performed in triplicate and a quadratic polynomial line was fit through the averages of these data points. All reactions and measurements were performed analogously to the batch reactions described in the main manuscript.

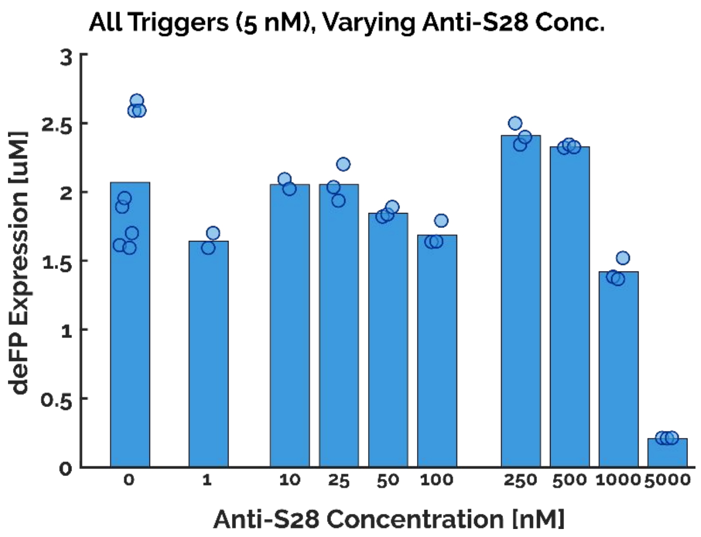

Figure S4: Endpoint deGFP expression levels of reactions with all three inputs present for a range of anti-σ^{28} concentrations. Expression levels do not decrease significantly until at least 1 µM of the anti-σ^{28} has been added to the reactions, however the signal is not turned 'OFF' until a concentration of 5 µM has been applied. All three weights were present in each reaction with the following weight pattern: weights 1 and 2: 0.6 nM and weight 3: 0.8 nM. Additionally, 12.5 nM of the deGFP reporter construct was added to each reaction. The height of the bars is the average of multiple experiments, indicated by the circles.

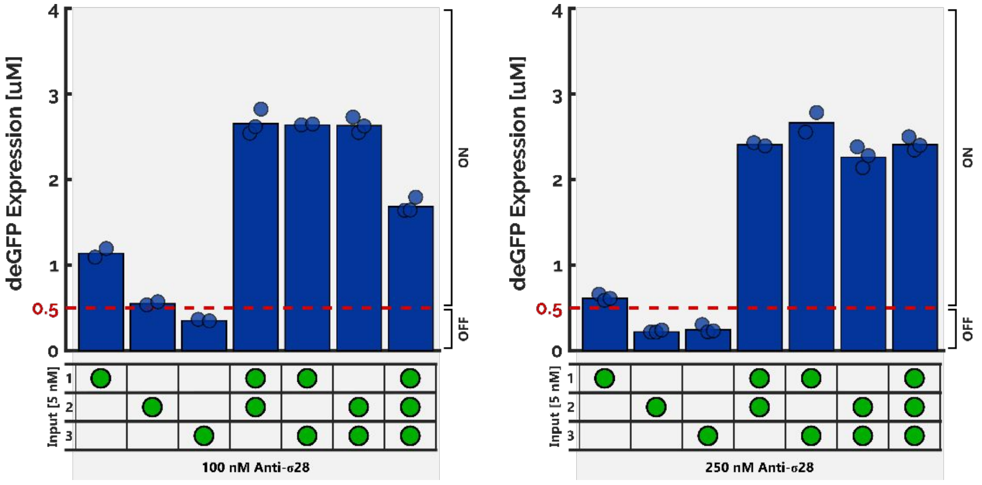

Figure S5: Endpoint deGFP expression levels of reactions with either one, two, or three inputs present, in the presence of 100 nM (left) or 250 nM (right) anti-σ^{28}. In both cases, at least one of the single input expression levels exceeds the ON/OFF threshold concentration of 0.5 µM. As such, these concentrations of anti-σ^{28} are insufficient to distinguish single input reactions from double or triple input reactions. All three weights were present in each reaction with the following weight pattern: weights 1 and 2: 0.6 nM and weight 3: 0.8 nM. Additionally, 12.5 nM of the deGFP reporter construct was added to each reaction.

DNA Construct	Sequence
pBEST_LinL2_F	ACATTTCCCCGAAAAGTGCCAC
pBEST_LinL2_R	TATCGTCTTGAGTCCAACCCGG
pBEST_GA_1_F	GATACCGCGCGACCCACGCTCACC
pBEST_GA_1_R	GTGGGTCGCGCGGTATCATTGCAGCAC
pBEST_GA_2_F	CCTCAGGTAGGATGTAGGCCCTCAAAGAGATTGGCGCGGTGCTGG
pBEST_GA_2_R	GGCCTACATCCTACCTGAGGCGTTACCGGACCAGAAGTTGTCCTGGC
Trigger 1	GATACACATAGAATCATGTGTATAACACTACTAAACTTCTATCATATTCAATCAC
Trigger 2	GAGTCTTCAAGATAATGAAGACTCTGGACATAGAACGTATTACACTCATAAGATA
Trigger 3	ACCGCAATGCGGAAATTGCGGTAATGAAATAGAAACAGAACAGCAGGGATAAACGAGATAGATAAGAT<br>AAGAATGAAATGAATAGAGGCGAATAGCATAACCCCTT
Switch 1	GATTGAATATGATAGAAGTTTAGTAGTAGACAATAGAACAGAGGAGATATTGATGACTACTAAACTA
Switch 2	GCTTATGAGTGTAATACGTTCTATGTCAGATTCAAGAACAGAGGAGATTGAAATGGACATAGAACGA
Switch 3	GTCTTATCTTATCTATCTCGTTTATCCCTGCATACAGAAACAGAGGAGATATGCAATGATAAACGAG
Linker (Follows switch, prior to protein)	ACCTGGCGGCAGCGCAAAAGTTG
P70a Promoter (OR2-OR1-Pr)	TGAGCTAACACCGTGCGTGTTGACAATTTTACCTCTGGCGGTGATAATGGTTGCA
P28a Promoter (Ptar)	CAAGCTTCAATAAAGTTTCCCCCCTCCTTGCCGATAACGAGATCAA
Terminator (T500)	CAAAGCCCGCCGAAAGGCGGGCTTTTCTGT
deGFP	GCTTATGAGTGTAATACGTTCTATGTCAGATTCAAGAACAGAGGAGATTGAAATGGACATAGAACGAA<br>ACCTGGCGGCAGCGCAAAAGTTGGAGCTTTTCACTGGCGTTGTTCCCATCCTGGTCGAGCTGGACGGC<br>GACGTAAACGGCCACAAGTTCAGCGTGTCCGGCGAGGGCGAGGGCGATGCCACCTACGGCAAGCTGAC<br>CCTGAAGTTCATCTGCACCACCGGCAAGCTGCCCGTGCCCTGGCCCACCCTCGTGACCACCCTGACCT<br>ACGGCGTGCAGTGCTTCAGCCGCTACCCCGACCACATGAAGCAGCACGACTTCTTCAAGTCCGCCATG<br>CCCGAAGGCTACGTCCAGGAGCGCACCATCTTCTTCAAGGACGACGGCAACTACAAGACCCGCGCCGA<br>GGTGAAGTTCGAGGGCGACACCCTGGTGAACCGCATCGAGCTGAAGGGCATCGACTTCAAGGAGGACG<br>GCAACATCCTGGGGCACAAGCTGGAGTACAACTACAACAGCCACAACGTCTATATCATGGCCGACAAG<br>CAGAAGAACGGCATCAAGGTGAACTTCAAGATCCGCCACAACATCGAGGACGGCAGCGTGCAGCTCGC<br>CGACCACTACCAGCAGAACACCCCCATCGGCGACGGCCCCGTGCTGCTGCCCGACAACCACTACCTGA<br>GCACCCAGTCCGCCCTGAGCAAAGACCCCAACGAGAAGCGCGATCACATGGTCCTGCTGGAGTTCGTG<br>ACCGCCGCCGGGATCTAA
σ^{28}	GCTTATGAGTGTAATACGTTCTATGTCAGATTCAAGAACAGAGGAGATTGAAATGGACATAGAACGAA<br>ACCTGGCGGCAGCGCAAAAGTTGAATTCACTCTATACCGCTGAAGGTGTAATGGATAAACACTCGCTG<br>TGGCAGCGTTATGTCCCGCTGGTGCGTCACGAAGCATTGCGCCTGCAGGTTCGACTGCCCGCGAGCGT<br>GGAACTTGACGATCTGCTACAGGCGGGCGGCATTGGGTTACTTAATGCCGTCGAACGCTATGACGCCC<br>TACAAGGAACGGCATTTACAACTTACGCAGTGCAGCGTATCCGTGGCGCTATGCTGGATGAACTTCGC<br>AGCCGTGACTGGGTGCCGCGCAGCGTGCGACGCAACGCGCGTGAAGTGGCACAGGCAATAGGGCAACT<br>GGAGCAGGAACTTGGCCGCAACGCCACGGAAACTGAGGTAGCGGAACGTTTAGGGATCGATATTGCCG<br>ATTATCGCCAAATGTTGCTCGACACCAATAACAGCCAGCTCTTCTCCTACGATGAGTGGCGCGAAGAG<br>CACGGCGATAGCATCGAACTGGTTACTGATGATCATCAGCGAGAAAACCCGCTACAACAACTACTGGA<br>CAGTAATCTGCGCCAGCGGGTGATGGAAGCCATCGAAACGTTGCCGGAGCGCGAAAAACTGGTATTAA<br>CCCTCTATTACCAGGAAGAGCTGAATCTCAAAGAGATTGGCGCGGTGCTGGAGGTCGGGGAATCGCGG<br>GTCAGTCAGTTACACAGCCAGGCTATTAAACGGTTACGCACTAAACTGGGTAAGTTATGA
pBest Plasmid	www.addgene.org/45779/

Table S1: DNA sequences of specific constructs used during this research. RBS sites and start codons required to translate the proteins are incorporated within the switch sequences. Protein sequences show a switch (2), a short linker, and the expressed protein sequence analogous to the manner in which they were implemented throughout this research. The hairpin forming sequences as well as the toehold sequence have also been indicated. The additional sequence to enable NOT-gate functionality on trigger 3 has also been indicated.

Supplementary Methods 1

A rudimentary model was implemented to predict the expression levels of the WSO, based on equation 1:

$$WSO = \alpha \cdot In1 \cdot Wt1 + \beta \cdot In2 \cdot Wt2 + \gamma \cdot In3 \cdot Wt3 \qquad \text{Eq. 1}$$

Herein, the WSO output is defined as the total sum of the product of all input (*In*) and weight (*Wt*) pairs and their respective scaling factors: α, β, and γ. Input and weight values are given as concentrations, whereas the scaling factors are dimensionless and serve to reflect a variety of biological process such as transcription and translation, whilst also reflecting the respective expression strengths of each input-weight pair.

Under batch conditions a range of WSO were conducted, with each reaction containing all three weights in a concentration matching that of the specified weight pattern (weight 1: 1 nM, weight 2: 5 nM, and weight 3: 7.5 nM). Subsequently 1, 2, or 3 of the inputs were provided to each reaction, such that all possible input combinations were tested at least twice. Following the batch experiments, the endpoint expression levels for each reaction were recorded.

Using the expression data obtained from the single input experiments, Eq 1. was fit to the experimental data using the nonlinear least squares solver native to Matlab: *lsqnonlin.*

An initial value of 0.1 was chosen for each of the three scaling factors and upper and lower bounds were set at 10^2 and 10^{-4} respectively. When fitting the function, the input and weight concentrations used to conduct the single input batch experiments were provided, alongside the experimentally determined expression levels of each experiment. The following function was then prepared to allow for fitting of the scaling factors.

```
for i = 1:size(endpointData,2)
        WSO = prodRates.*trig(i,:).*swit(i,:);
        TotWSO = sum(WSO);
        modelWSO(i) = TotWSO;
end

diff = endpointData - modelWSO;
```

Here, endpoint data contains the experimentally determined expression data for the single input reactions, *'prodRates'* is a vector of the three scaling factors, *'trig'* is a vector indicating the concentration of each of the possible inputs, and '*swit*' is a vector indicating the concentrations of each of the weights present.

For each of the single input experiments, the WSO output (*'TotWSO'*) is determined for a specific set of scaling factors. Once the model output for each unique set of inputs has been determined for the specific set of scaling factors (stored in *'modelWSO')* the model data can be compared to the experimental data to provide a vector containing the differences between the experimental and computational WSO outcomes: *'diff'.*

The *lsqnonlin* function iteratively performs the above computation, repeatedly determining the difference vector *'diff'* whilst varying the scaling factors until a minimum has been found. At this point the optimal scaling factors are stored. By performing the fit in this manner, for multiple identical inputs with slight variations in the experimental output, scaling factors can be found such that the model output for single input reactions is approximately equal to the average of the experimental results for identical inputs.

With the previously determined scaling factors, Eq 1. can be used to predict the expression levels of multi-input reactions. Ideally, the multi-input reactions would scale with the single input reactions such that the expression levels of a two input reaction are equal to the sum of the expression levels of two single input reactions, however it has been shown that this is not the case. However, due to the rudimentary nature of the model (Eq. 1) this is how the expression levels are computationally predicted, with the model ignoring any resource competition or cross talk between DNA or RNA species. As such the model predictions exceed the values of the experimental reactions significantly as shown in **Figure 2c**.

Supplementary Methods 2

The prediction of expression levels for the coupled WSO occurred in a similar fashion to those described in **Supplementary Methods 1**, the major difference being the inclusion of a second equation:

$$Output = WSO \cdot Reporter \qquad \text{Eq. 2}$$

Here, the report expression levels (*'Output'*) are determined as the product of the WSO output (*'WSO'*) and the concentration of the reporter construct (*'Reporter'*). The *'WSO'* is determined using Eq. 1 and the concentration of the reporter construct is set during the experiments (15 nM). Analogously to the model predictions of the direct expression WSO, the model scaling factors were first determined by fitting the model to the single input expression data using the *lsqnonlin* non-linear least squares solver.

Experimentally, batch experiments were performed whereby all possible input combinations (single, double, and triple) were add to reactions, all of which contained 15 nM of the reporter construct and all the weights in concentrations matching those determined in the weight pattern: weights 1 and 2: 0.6 nM, weight 3: 0.8 nM. All inputs were provided at a concentration of 5 nM.

When fitting the model to the single input data, the initial values of all scaling factors was set to 0.1. The upper and lower limits were determined as 10^{2} and 10^{-4} respectively. For each of the single input conditions, the model was provided with multiple experimentally determined data points obtained from triplicate reactions. As such, the scaling factors determined in the fitting process approximate the average of the experimental triplicates. During the fitting process, a similar function was used to determine the difference between the experimental data and the model predictions for a specified set of scaling factors. Crucially however, the WSO output value was now multiplied by the reporter concentration to obtain the final model prediction:

```
for i = 1:size(endpointData,2)
        S28_Per_Swit = prodRates(1:3).*trig(i,1:3).*swit(i,1:3);
        Tot_S28 = sum(S28_Per_Swit);
        EGFP_Prod = Tot_S28*outputConc;
        modelWSO(i) = EGFP_Prod;
end

diff = endpointData - modelWSO;
```

Here *'S28_Per_Swit'* comprises the WSO output, with *'EGFP_Prod'* representing the model predicted reporter expression level. All other variables are analogous to those used in **Supplementary Methods** 1. Again, using the *lsqnonlin* function, the above function was iterated until a minimum difference was found between the experimental endpoint data (*'endpointData'*) and the model predictions (*'modelWSO'*), at which point the scaling factors were recorded and used to fill in equations 1 and 2, to provide the model predicted expression levels for the coupled WSO. As opposed to the direct expression WSO the model predictions for the coupled WSO closely match the experimentally determined values, with only a slight overestimation of the actual expression levels.

The decision was made not to include an additional variable to represent the biological processes in Eq 2. as has been done in Eq. 1. This results from the fact that the output of Eq. 2 is simply the output of Eq. 1 multiplied by the reporter concentration. Since Eq. 1 is merely a summation of products, the addition of an additional variable in Eq. 2 would not affect the model fit or predictions, as the scaling factors found in Eq. 1 would be normalized to the additional variable.

DNA Plasmid Sequence of the pBest vector: **pBEST-P70a-Sw1-S28**

```
1      ttaccaatgcttaatcagtgaggcacctatctcagcgatctgtctatttcgttcatccata
62     gttgcctgactccccgtcgtgtagataactacgatacgggagggcttaccatctggcccca
123    gtgctgcaatgataccgcgacccacgctcaccggctccagatttatcagcaataaacca
184    gccagccggaagggccgagcgcagaagtggtcctgcaactttatccgcctccatccagtct
245    attaattgttgccgggaagctagagtaagtagttcgccagttaatagtttgcgcaacgttg
306    ttgccattgctacaggcatcgtggtgtcacgctcgtcgtttggtatggcttcattcagctc
367    cggttcccaacgatcaaggcgagttacatgatcccccatgttgtgcaaaaaagcggttagc
428    tccttcggtcctccgatcgttgtcagaagtaagttggccgcagtgttatcactcatggtta
489    tggcagcactgcataattctcttactgtcatgccatccgtaagatgcttttctgtgactgg
550    tgagtactcaaccaagtcattctgagaatagtgtatgcggcgaccgagttgctcttgcccg
611    gcgtcaatacgggataataccgcgccacatagcagaactttaaaagtgctcatcattggaa
672    aacgttcttcggggcgaaaactctcaaggatcttaccgctgttgagatccagttcgatgta
733    acccactcgtgcacccaactgatcttcagcatcttttactttcaccagcgtttctgggtga
794    gcaaaaacaggaaggcaaaatgccgcaaaaaagggaataagggcgacacggaaatgttgaa
855    tactcatactcttcctttttcaatattattgaagcatttatcagggttattgtctcatgag
916    cggatacatatttgaatgtatttagaaaaataaacaaataggggttccgcgcacatttccc
977    cgaaaagtgccacctgacgtctaagaaaccattattatcatgacattaacctataaaaata
1038   ggcgtatcacgaggccctttcgtcttcaagaattctggcgaatcctctgaccagccagaaa
1099   acgacctttctgtggtgaaaccggatgctgcaattcagagcggcagcaagtgggggacagc
1160   agaagacctgaccgccgcagagtggatgtttgacatggtgaagactatcgcaccatcagcc
1221   agaaaaccgaattttgctgggtgggctaacgatatccgcctgatgcgtgaacgtgacggac
1282   gtaaccaccgcgacatgtgtgtgctgttccgctgggcatgccaggacaacttctggtccgg
1343   taacgcattgagctaacaccgtgcgtgttgacaattttacctctggcggtgataatggttg
1404   cagattgaatatgatagaagtttagtagtagacaatagaacagaggagatattgatgacta
1465   ctaaactaaacctggcggcagcgcaaaagttgaattcactctataccgctgaaggtgtaat
1526   ggataaacactcgctgtggcagcgttatgtcccgctggtgcgtcacgaagcattgcgcctg
1587   caggttcgactgcccgcgagcgtggaacttgacgatctgctacaggcgggcggcattgggt
1648   tacttaatgccgtcgaacgctatgacgccctacaaggaacggcatttacaacttacgcagt
1709   gcagcgtatccgtggcgctatgctggatgaacttcgcagccgtgactgggtgccgcgcagc
1770   gtgcgacgcaacgcgcgtgaagtggcacaggcaatagggcaactggagcaggaacttggcc
1831   gcaacgccacggaaactgaggtagcggaacgtttagggatcgatattgccgattatcgcca
1892   aatgttgctcgacaccaataacagccagctcttctcctacgatgagtggcgcgaagagcac
1953   ggcgatagcatcgaactggttactgatgatcatcagcgagaaaacccgctacaacaactac
2014   tggacagtaatctgcgccagcgggtgatggaagccatcgaaacgttgccggagcgcgaaaa
2075   actggtattaaccctctattaccaggaagagctgaatctcaaagagattggcgcggtgctg
2136   gaggtcggggaatcgcgggtcagtcagttacacagccaggctattaaacggttacgcacta
2197   aactgggtaagttatgaacgccactcgagagtcgaccaaagcccgccgaaaggcgggcttt
2258   tctgtgccggcatgataagctgtcaaacatgagaattacaacttatatcgtatggggctga
2319   cttcaggtgctacatttgaagagataaattgcactgaaatctagaaatattttatctgatt
2380   aataagatgatcttcttgagatcgttttggtctgcgcgtaatctcttgctctgaaaacgaa
2441   aaaaccgccttgcagggcggtttttcgaaggttctctgagctaccaactctttgaaccgag
2502   gtaactggcttggaggagcgcagtcaccaaaacttgtcctttcagtttagccttaaccggc
2563   gcatgacttcaagactaactcctctaaatcaattaccagtggctgctgccagtggtgcttt
2624   tgcatgtctttccgggttggactcaagacgatagttaccggataaggcgcagcggtcggac
2685   tgaacggggggttcgtgcatacagtccagcttggagcgaactgcctacccggaactgagtg
2746   tcaggcgtggaatgagacaaacgcggccataacagcggaatgacaccggtaaaccgaaagg
2807   caggaacaggagagcgcacgagggagccgccagggggaaacgcctggtatctttatagtcct
2868   gtcgggtttcgccaccactgatttgagcgtcagatttcgtgatgcttgtcaggggggcgga
2929   gcctatggaaaaacggctttgccgcggccctctcacttccctgttaagtatcttcctggca
2990   tcttccaggaaatctccgccccgttcgtaagccatttccgctcgccgcagtcgaacgaccg
3051   agcgtagcgagtcagtgagcgaggaagcggaatatatcctgtatcacatattctgctgacg
3112   caccggtgcagcctttttctcctgccacatgaagcacttcactgacaccctcatcagtgc
3173   caacatagtaagccagtatacactccgctagggtcatgagattatcaaaaaggatcttcac
3234   ctagatcctttaaattaaaaatgaagttttaaatcaatctaaagtatatatgagtaaact
3295   tggtctgacag
```

Legend:

Ampicillin Resistance	(1-861)
P70a promoter	(1351-1406)
Switch	(1407-1472)
Toehold sequence	(1408-1422)
Hairpin sequences	(1423-1433,1437-1441,1453-1457,1461-1471)
RBS site	(1445-1452)
Start codon	(1458-1460)
Linker	(1473-1496)
S28 Protein	(1497-2210)
Terminator Sequence	(2530-2262)
Primer Binding Sites	(968-989,2636-2658)
